# Newly Designed Primers for the Sequencing of the *inlA* Gene of Lineage I and II *Listeria monocytogenes* Isolates

**DOI:** 10.3390/ijms232214106

**Published:** 2022-11-15

**Authors:** Giulia Magagna, Guido Finazzi, Virginia Filipello

**Affiliations:** 1 Food Safety Department, Istituto Zooprofilattico Sperimentale della Lombardia e dell’Emilia Romagna (IZSLER), Via A. Bianchi 9, 25124 Brescia, Italy; 2 Centro di Referenza Nazionale per i Rischi Emergenti in Sicurezza Alimentare—CRESA, Via A. Bianchi 9, 25124 Brescia, Italy

**Keywords:** *Listeria monocytogenes*, internalin A, PMSC, primers

## Abstract

*Listeria monocytogenes* is a major human foodborne pathogen responsible for listeriosis. The virulence factor Internalin A (inlA) has a key role in the invasion of *L. monocytogenes* into the human intestinal epithelium, and the presence of premature stop-codons (PMSC) mutations in the *inlA* gene sequence is correlated with attenuated virulence. The inlA sequencing process is carried out by dividing the gene into three sections which are then reassembled to obtain the full gene. The primers available however were only able to entirely amplify the lineage II isolates. In this study, we present a set of new primers which allow *inlA* sequencing of isolates belonging to both lineages, since lineage I isolates are the ones most frequently associated to clinical cases. Using newly designed primers, we assessed the presence of inlA PMSCs in food, food processing environments and clinical isolates.

## 1. Introduction

*Listeria monocytogenes* is a ubiquitous bacterium which colonizes several ecological niches, such as soil, water, food, and food processing plants [1,2]. In humans, it is responsible for inducing listeriosis, which can induce various clinical syndromes such as gastroenteritis in healthy individuals, or sepsis, meningitis, encephalitis in immunocompromised patients, the elderly, and neonates, and miscarriage or stillbirth in pregnant women [3,4].

*L. monocytogenes* comprises 4 phylogenetic lineages and 13 serotypes [4,5]. Serotypes 1/2b and 4b, belonging to lineage I, are the most frequent in clinical cases, while serotypes 1/2a and 1/2c, belonging to lineage II, are more frequently observed in food and food processing environments [6].

*L. monocytogenes* exhibits different virulence factors, including the surface protein internalin A (inlA), which mediates the internalization of the bacterium in human intestinal cells through interaction with the E-cadherin receptor [7]. Several studies have reported that the *inlA* gene can carry premature stop codon (PMSC) mutations leading to a truncated form of the protein, which is secreted rather than being anchored to the bacterial cell wall, which is associated with its attenuated invasive capacity and to the hypovirulent phenotype of the bacterium [8,9,10,11]. Indeed, PMSCs in *inlA* occur in 35–45% of food and food-related isolates, but they are rarely observed in listeriosis clinical cases [11,12,13,14]. In addition, the truncated protein may influence stress tolerance, such as cold and desiccation sensibility, adhesion capability, and biofilm formation, giving a possible explanation for the persistence of certain *L. monocytogenes* strains in food processing environments [15,16]. Nowadays, a total of 29 PMSC mutation types have been found (https://bigsdb.pasteur.fr/listeria/; accessed on 7 June 2022).

In the literature three different approaches are reported to evaluate the presence of PMSCs in the *inlA* gene. The first consists of four pairs of primers which cover the whole gene of isolates belonging to both lineages; the second one is based on a pair of primers for a real-time PCR assay which detects three specific PMSCs; the last approach uses three pairs of primers that have been tested on lineage II isolates only [17,18,19]. The latter has several advantages, with the major one being represented by having to sequence fewer fragments, which consequently decreases the cost for reagents, the time for the assembly and analysis of the *inlA* sequence, and the possibility of identifying all 29 PMSC mutations. The major drawback of this protocol is that sequencing of the whole gene is possible only for lineage II isolates.

Therefore, to overcome the issues linked to *inlA* amplification of lineage I isolates, the aim of the present report is to provide an updated primer set for the sequencing of the *inlA* gene of *L. monocytogenes* isolates belonging to both lineage I and lineage II to evaluate the presence of PMSCs.

## 2. Results

**Figure 1:**
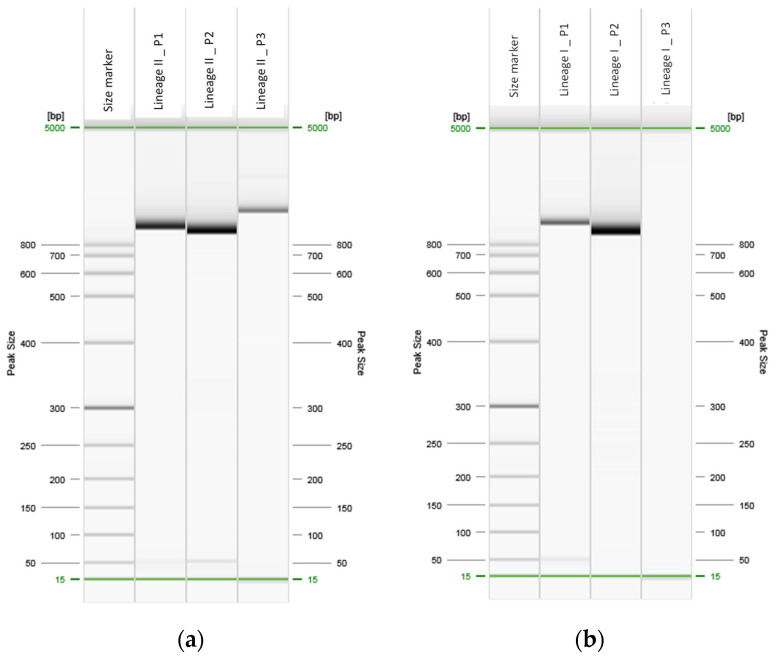

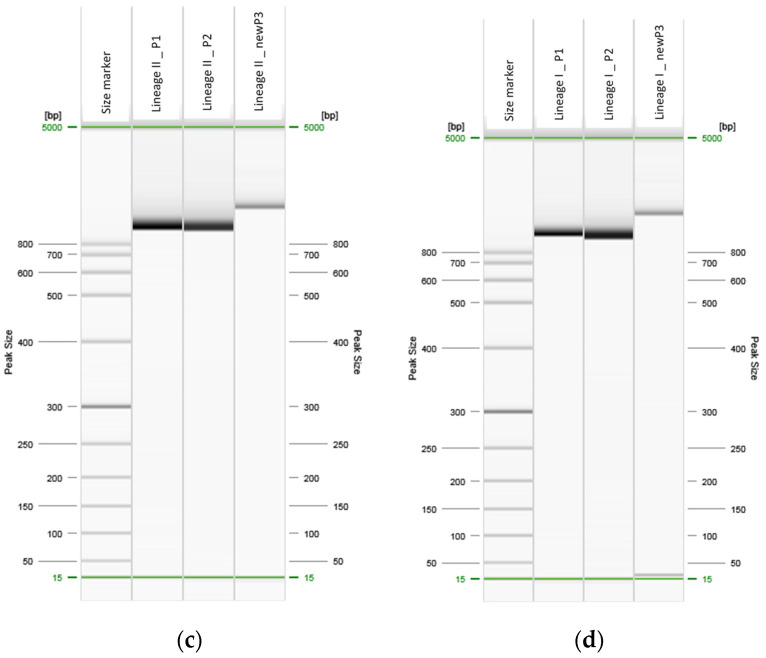
Digital image of capillary electrophoresis of *inlA* amplification: (**a**) an isolate belonging to lineage II, and (**b**) an isolate belonging to lineage I using the primers set designed by Gelbíčová et al. [19]; (**c**) an isolate belonging to lineage II, and (**d**) an isolate belonging to lineage I using the third fragment of the newly designed primers.

Primers found in the literature previously described by Gelbíčová et al. [19] allowed *inlA* sequencing of isolates belonging to lineage II (Figure 1a) and the sequencing of the first two parts of the gene of isolates belonging to lineage I (Figure 1b and Figure S1a).

**Figure 2:**
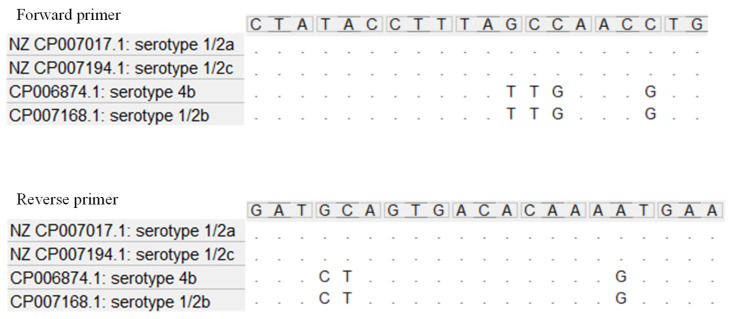
Forward and reverse sequences of the annealing region of the third set of primers by Gelbíčová et al. [19] in the four major serotypes of lineage I and II.

Indeed, the sequencing of the whole gene of lineage I isolates (serotype 4b and serotype 1/2b) is not possible due to the presence of mismatches in the annealing region of the third pair of primers (Figure 2 and Figure S2).

Considering this, new primers were designed using degenerate bases (Y, V, R, S) to allow for the sequencing of the third *inlA* fragment in isolates belonging to both lineages (Figure 1c,d and Figure S1b).

The new primers from all 40 isolates, of which 17 isolates (42%) were collected from food, 10 isolates (25%) were collected from food-related environments, and 13 isolates (33%) were collected from clinical cases, were successfully sequenced. Among the sequenced isolates, 75% (n = 30) presented a full-length inlA, while 25% (n = 10) exhibited PMSCs. PMSCs were found only in food and food processing plants isolates belonging to lineage II, in particular in 41% (n = 7) of food isolates and in 30% (n = 3) of environmental isolates. All clinical isolates (n = 13) presented a full-length inlA.

## 3. Discussion

In this study we designed a new set of primers able to sequence isolates belonging to both lineage I and II in only three fragments. The analysis of lineage I isolates is crucial because they are mainly related to clinical cases and are found less often in food and environmental isolates. In fact, previous studies have demonstrated that strains carrying *inlA* PMSCs are rarely found in lineage I, and they are usually collected from food and environmental sources rather than clinical cases [20,21]. In light of this, *inlA* sequencing of isolates belonging to lineage I can be important in order to increase knowledge on the effect of PMSCs on attenuated virulence.

Unlike the primers previously designed, this set of primers enables full *inlA* amplification of isolates belonging to both lineages and has the advantage of reducing costs and analysis time as three fragments are generated instead of four. Moreover, while whole genome sequencing (WGS) is becoming a common typing approach, a Sanger sequencing protocol for *inlA* sequencing can still be convenient and straightforward for those settings in which WGS is not yet routinely applied.

It is well-known that isolates from lineage I are generally more virulent than isolates belonging to lineage II. Indeed, our results are in line with the literature as most clinical isolates linked to invasive disease belonged to lineage I [22]. However, as previously reported, 56% (n = 15) of food-origin isolates and environmental isolates were from lineage II underling that this lineage is more related to possible food contaminations than to clinical cases [23]. In the set of isolates selected for this study, *inlA* PMSCs were found only in *L. monocytogenes* lineage II isolates, confirming a possible reason for the high prevalence of this lineage in food-correlated products and for the low frequency among clinical cases [11]. These findings are consistent with previous studies carried out in France, the United States, and Italy, in which a significant proportion (30–40%) of *L. monocytogenes* isolates from food and from food processing plants presented a truncated inlA, while all isolates from human listeriosis cases had a full-length protein [11,24,25].

## 4. Materials and Methods

### 4.1. Isolates Collection

Among *L. monocytogenes* isolates previously typed with MLST and collected from food, food processing environments, and clinical cases during routine surveillance between 2013–2022 in Lombardy, 40 were selected for sequencing to obtain a set of isolates representative of the different lineages (20 isolates belonging to lineage I and 20 isolates of lineage II), sequence types (STs), and origin (food, environment, and clinical) (Table 1).

### 4.2. Primers

Amplification of the whole *inlA* (2400 bp) was carried out using three pairs of primers [19]. For the first two parts of *inlA*, primers previously detailed by Gelbíčová et al. [19], were used. The third region of *inlA* was amplified using both primers of Gelbíčová et al. [19], and new primers modified as follow: Snew_3F: YTATACCTTTAVCCAAYCTG, Snew_3R: TTCAYTTTGTGTCACTRSATC (Sigma-Aldrich, St. Louis, MO, USA) (Table 2). For the design of new primers, the annealing regions of the third forward and reverse primer pair described by Gelbíčová et al. [19] were aligned using software MEGA version 6 (Molecular Evolutionary Genetics Analysis Version 6.0) [26] with reference sequences belonging to different serotypes: serotype 1/2a (NZ_CP007017.1), serotype 1/2c (NZ_CP007194.1), serotype 4b (CP006874.1), and serotype 1/2b (CP007168.1) (Figure 2).

### 4.3. PCR Assay

PCR reactions contained: HotStarTaq Master Mix Kit (1X) (Qiagen, Hilden, Germany), DNase-RNase-free water, and forward and reverse primers (0.6 µM). The cycling conditions were as follows: a denaturation step at 96 °C for 15 min and 35 cycles of 30 s at 94 °C, 90 s at 55 °C, and 90 s at 72 °C and a final extension step at 72 °C for 10 min. The successful amplification of PCR products was visualized with capillary electrophoresis on a QIAxcel Advanced System (Qiagen, Hilden, Germany). The PCR products were purified with ExoSAP-IT™ Express PCR Product Cleanup Reagent (Thermo Fisher Scientific, Waltham, MA, USA) according to the instructions. Cycle sequencing, for forward and reverse sequences, was performed using BigDye™ Terminator v1.1 Cycle Sequencing (Thermo Fisher Scientific, Waltham, MA, USA) in a GeneAmp^®^ PCR System 9700 (Thermo Fisher Scientific, Waltham, MA, USA). The products were then purified using the BigDye Xterminator™ Purification Kit (Thermo Fisher Scientific, Waltham, MA, USA) according to the instructions. Then, the samples were run on an Applied Biosystems Seqstudio Genetic Analyzer (Thermo Fisher Scientific, Waltham, MA, USA) with the long_BDX run module. After sequencing, the three consensus sequences for each sample were aligned and assembled in a single sequence of 2400 bp with software MEGA version 6 (Molecular Evolutionary Genetics Analysis Version 6.0) [26] using the *inlA* sequence as a template with accession number MG922918.1.

## 5. Conclusions

The assessment of *L. monocytogenes* pathogenicity is a discussed concern and *inlA* analysis has a key role to play in the virulence evaluation. Given the high prevalence of *inlA* mutations in food and environmental isolates rather than in clinical isolates, the detection of PMSCs may provide information to support studies that assess virulence variability among isolates.

In light of this, the set of primers presented in this study allows for the efficient amplification and, consequently, the sequencing of the entire *inlA* gene of *L. monocytogenes* isolates belonging to both lineage I, and lineage II and may prove to be useful for assessing the pathogenic potential of *L. monocytogenes* isolates from different origins.

## Figures and Tables

**Table 1:** Forty *L. monocytogenes* isolates selected for *inlA* sequencing considering lineage, ST, origin, and source.

GenBank Accession Number	Isolation Year	Lineage	ST	Origin	Source	Clinical Syndrome
OP686908	2020	I	1	Food	Meat	/
OP686909	2020	I	1	Clinical	Blood	Sepsis
OP686910	2020	I	1	Food	Cheese	/
OP686912	2020	I	1	Clinical	Blood	Sepsis
OP686923	2021	I	1	Food	Meat	/
OP686924	2021	I	1	Clinical	Blood	Sepsis
OP686926	2021	I	1	Clinical	Placenta	Maternal-neonatal
OP686918	2020	I	2	Food	Other	/
OP686921	2021	I	2	Food	Cheese	/
OP686928	2021	I	2	Clinical	Cerebrospinal fluid	Meningitis
OP686913	2020	I	3	Environmental	Meat	/
OP686920	2020	I	3	Food	Fish	/
OP686914	2020	I	3	Clinical	Blood	Sepsis
OP686911	2020	I	6	Clinical	Blood	Sepsis
OP686929	2021	I	6	Clinical	Blood	Sepsis
OP686917	2020	II	7	Environmental	Meat	/
OP686906	2019	II	8	Clinical	Blood	Sepsis
OP686907	2019	II	8	Environmental	Grocery store	/
OP686919	2020	II	8	Clinical	Blood	Sepsis
OP686925	2021	II	8	Clinical	Blood	Sepsis
OP686944	2019	II	9	Food	Meat	/
OP686936	2020	II	9	Food	Other	/
OP686935	2020	II	9	Environmental	Grocery store	/
OP686930	2021	II	26	Clinical	Blood	Sepsis
OP686905	2020	II	37	Food	Salami	/
OP686927	2021	II	37	Clinical	Blood	Sepsis
OP686940	2020	II	121	Environmental	Meat	/
OP686941	2020	II	121	Food	Meat	/
OP686942	2020	II	121	Food	Meat	/
OP686943	2020	II	121	Food	Meat	/
OP686939	2020	II	121	Food	Meat	/
OP686932	2020	II	155	Environmental	Meat	/
OP686915	2020	II	204	Environmental	Dairy	/
OP686922	2021	I	217	Environmental	Meat	/
OP686916	2021	I	217	Food	Milk	/
OP686933	2021	I	288	Food	Cheese	/
OP686934	2021	I	288	Environmental	Meat	/
OP686937	2013	II	325	Environmental	Dairy	/
OP686938	2015	II	325	Food	Cheese	/
OP686931	2020	I	330	Food	Meat	/

**Table 2:** Primers detailed by Gelbíčová et al. [19] and primers designed in this study.

Name	Forward 5′-3′ Sequence	Reverse 5′-3′ Sequence	Position (bp)
S_1	GATATCACTAAACGGCTCC	TAGTTTTGTTAGACCCGACA	(−170)–872
S_2	TAAATCGGCTAGAACTATCCA	GTCAATAAATTCCCAGCTTC	497–1540
S_3	CTATACCTTTAGCCAACCTG	TTCATTTTGTGTCACTGCATC	1410–(+218)
Snew_3	YTATACCTTTAVCCAAYCTG	TTCAYTTTGTGTCACTRSATC	1410–(+218)

## Data Availability

Sequences generated in this study have been submitted to GenBank (see Table 1 for references).

## Supplementary Materials

The following supporting information can be downloaded at: https://www.mdpi.com/article/10.3390/ijms232214106/s1.
