# Supplementary material for: Newly Designed Primers for the Sequencing of the inlA Gene of Lineage I and II Listeria monocytogenes Isolates
Source: Int J Mol Sci. 2022 Nov 15;23(22):14106. doi: 10.3390/ijms232214106 (PMC9698914; doi:10.3390/ijms232214106)
Supplement: Supplementary file 1 [file ijms-23-14106-s001.zip › ijms-1930031-supplementary.pdf]

Electronic Supplementary Materials

Newly Designed Primers for the Sequencing of *inlA* gene of lineage I and II *Listeria monocytogenes* isolates

Giulia Magagna, Guido Finazzi and Virginia Filipello

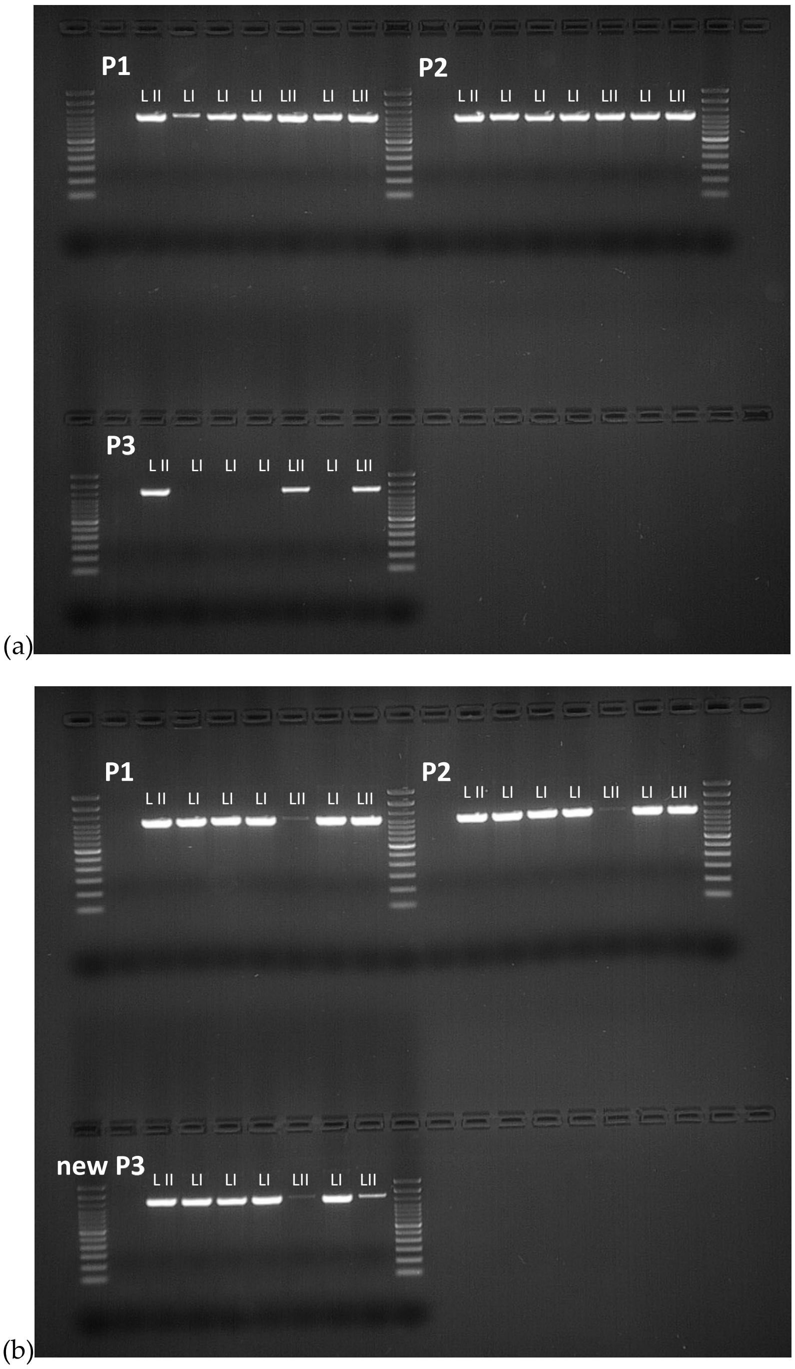

(a) (b)

Figure S1. Gel electrophoresis image of *inlA* amplification of 7 additional *L. monocytogenes* isolates belonging to lineage I and II employing (a) the three primers set designed by Gelbíčová *et al.* [19], and (b) for the third fragment, the newly designed primers.

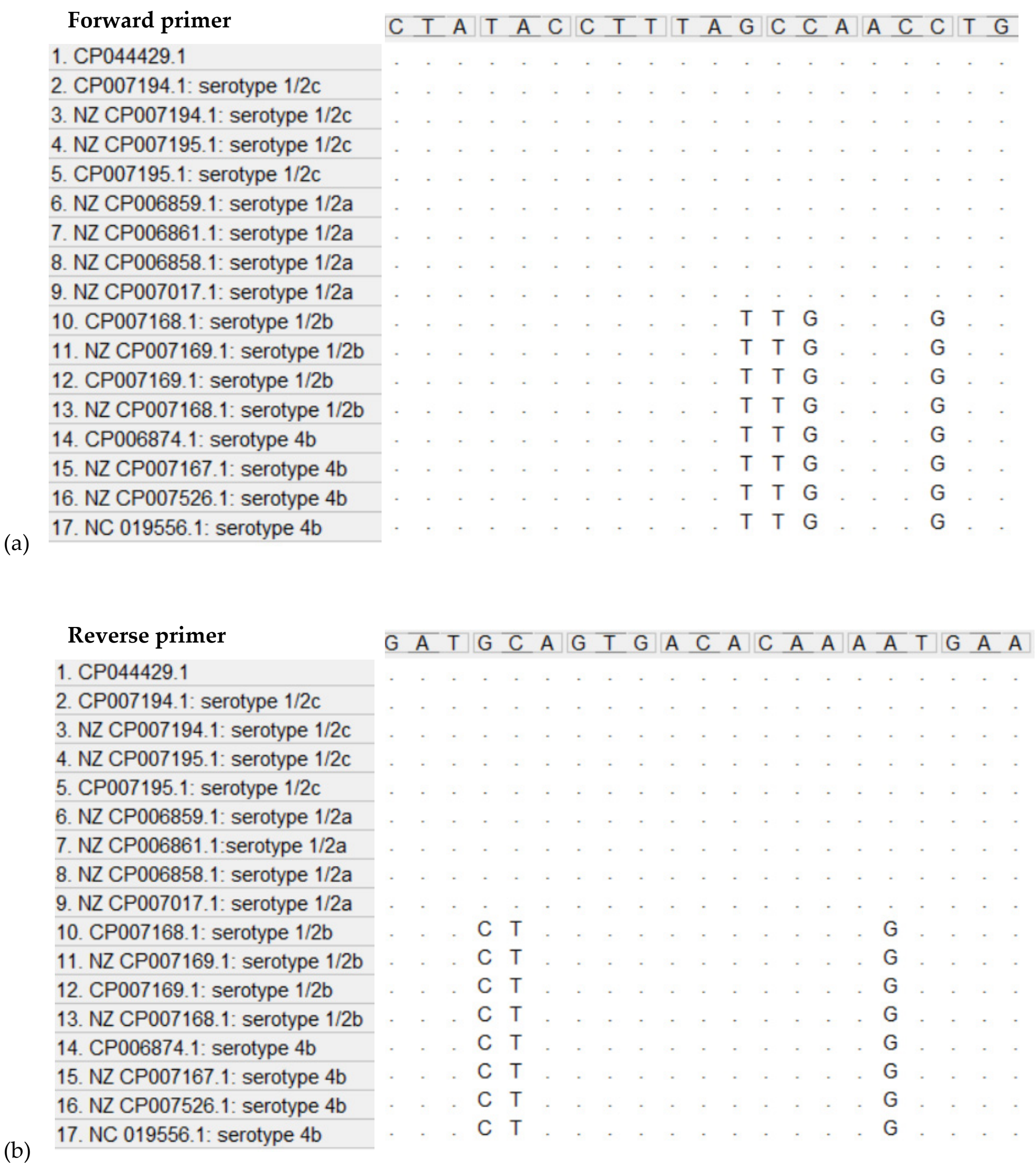

Figure S2. Forward (a) and reverse (b) sequences of the annealing region of the third set of primers designed by Gelbíčová *et al.* [19] of 16 reference isolates representing the four major serotypes of *L. monocytogenes* lineages I and II.
